# A Novel Detection Model and Its Optimal Features to Classify Falls from Low- and High-Acceleration Activities of Daily Life Using an Insole Sensor System

**DOI:** 10.3390/s18041227

**Published:** 2018-04-17

**Authors:** Benjamin Cates, Taeyong Sim, Hyun Mu Heo, Bori Kim, Hyunggun Kim, Joung Hwan Mun

**Affiliations:** 1 Department of Bio-Mechatronic Engineering, College of Biotechnology and Bioengineering, Sungkyunkwan University, 2066 Seobu-ro, Jangan-gu, Suwon, Gyeonggi 16419, Korea; bcates@skku.edu (B.C.); tysim@skku.edu (T.S.); hhmoo91@skku.edu (H.M.H.); 2 Department of Research and Development, Biomaterial Team, Medical Device Development Center, KBIO HEALTH, 123 Osongsaengmyung-ro, Osong-eub, Heungdeok-gu, Cheongju, Chungbuk 28160, Korea; borkim@kbiohealth.kr

**Keywords:** fall detection, high acceleration activities, insole sensor system, machine learning

## Abstract

In order to overcome the current limitations in current threshold-based and machine learning-based fall detectors, an insole system and novel fall classification model were created. Because high-acceleration activities have a high risk for falls, and because of the potential damage that is associated with falls during high-acceleration activities, four low-acceleration activities, four high-acceleration activities, and eight types of high-acceleration falls were performed by twenty young male subjects. Encompassing a total of 800 falls and 320 min of activities of daily life (ADLs), the created Support Vector Machine model’s Leave-One-Out cross-validation provides a fall detection sensitivity (0.996), specificity (1.000), and accuracy (0.999). These classification results are similar or superior to other fall detection models in the literature, while also including high-acceleration ADLs to challenge the classification model, and simultaneously reducing the burden that is associated with wearable sensors and increasing user comfort by inserting the insole system into the shoe.

## 1. Introduction

Falls are the leading cause of injury and injury death for the elderly population [1]. 30% of people that are 65 and over fall at least one time per year [2]. In 2015, the cost of falls to Medicare alone exceeded $31 billion [3]. Of particular concern to the medical community is the “long lie”; the condition in which a fallen patient is unable to get up and remains in a severely injured or unconscious state for a prolonged period of time awaiting help [4]. Receiving immediate help following a fall decreases the likelihood of hospitalization by 26% and reduces the chance of death by over 80% [5]. Even a fear of falling is a major inhibitor to physical activity [6], and the resulting decrease in activity leads to other deleterious health effects, which affect both the mind and the body [7]. Due to a significant increase in the elderly population [8], rising healthcare costs [3], and the potential for serious injury or even death, early, automatic fall event detection is a growing and necessary field of study.

There exist three primary methods for automatic fall detection: Cameras, other ambient devices, and on-body sensors. An algorithm utilizing a single camera distinguishes a human from the static background and measures the rapid changes in shape to detect falls as opposed to normal activities [9]. Dual Kinect cameras were used to establish a comprehensive dataset of simulated falls [10]. Kinect’s infrared sensor was used to detect falls by measuring the subject’s velocity based on changes in parameters to the three-dimensional (3D) bounding box [11]. A floor mat equipped with vibration sensors was implemented to detect falls out of bed and falls in the bathroom [12,13]. These camera-based and ambient device-based fall detection methods suffer from the limitation of line-of-sight and finite area conditions under which they can identify a fall. As a result of these limitations, on-body sensors, in particular, Inertial Measurement Units (IMUs), consisting of accelerometers and/or gyrometers, have been implemented with varying levels of success to detect falls and classify the activities of daily life.

Falling detection methods incorporating IMUs use either threshold [1,4,12,14,15,16] or machine learning methods [17,18,19]. Threshold methods identify a dividing threshold, primarily a derived feature called the Sum Vector Magnitude, which sufficiently separates the high acceleration magnitude’s of falls from the low-acceleration activities of daily life (ADLs), like walking, sitting, lying, etc., [1]. Threshold methods, although being low in computational complexity [15], are unable to generalize between diverse subjects and different types of falls [20]. Additionally, the thresholds that are derived from simulated falls often are not applicable to elderly falls [16] or other real-life falls [21]. A machine learning approach can overcome some of the lack of generalizability that is associated with threshold methods. Aziz et al. utilized seven IMUs that were attached to the ankles, thighs, waist, sternum, and head to distinguish near falls from low-acceleration ADLs with a five IMU combination, demonstrating 100% sensitivity and specificity, and a single sensor sensitivity of at least 80% [17]. Ozdemir et al. attached six IMUs via straps to the subjects’ chest, head, waist, wrist, thigh, and ankle to distinguish falls from low-acceleration ADLs with 99% accuracy [18]. Liu et al. proposed a machine learning algorithm for a single IMU system that is attached to the waist that distinguished falling events from low-acceleration ADLs, however had false positives that are associated with stair descension that this research does not have, as in addition, running or jumping activities were excluded [19]. The authors primarily identified low-acceleration ADLs (i.e., sitting, standing, walking, lying, and squatting) and although they did consider ascending and descending stairs, high false positive fall rates were observed. In addition, they did not consider running, jumping, or any other activities that are associated with high accelerations that a fall detection model could confuse with high-acceleration falls. Higher fall rates are strongly correlated with vigorous activities, like running, jumping, and climbing and descending stairs, in all age and gender groups except for elderly women [22]. Because falling events have associated high accelerations, it is important for a fall detection model to distinguish high-acceleration falls from high-acceleration ADLs [18].

Force-Sensitive Resistors (FSRs) are sensors that exhibit a decrease in resistance as the applied force is increased [23]. This resistance can be measured to calculate the amount of force that was applied on the sensor. They are often used in gait analysis [24]. With respect to fall classification, FSRs have been shown to be useful as an on/off switch [25]. FSR sensors can be extended to falling event detection by creating features that are useful for distinguishing high-acceleration ADLs and high-acceleration falls. Another strong benefit of FSRs is that they can be implemented in an insole alongside an IMU to create a single insole system. Multi-sensor systems attached across the body have proven to have higher performance than single-sensor systems in both the falling [18] and activity detection fields [26], but due to complexity and discomfort, they are impractical for everyday life. However, an insole system overcomes this limitation by creating a multi-sensor system in a single location. An insole system offers the benefits of simple integration with everyday footwear; it reduces the burden and conspicuousness associated with activity monitoring and facilitates everyday use, thus improving wearer compliance [27].

In order to distinguish falls from not only low-acceleration ADLs, but also high-acceleration ADLs, a novel fall detection model was developed. Additionally, in the process, the optimal features were described for the novel fall detection model. In this study, falls (walking fall, walking and stumbling fall, running fall, running and stumbling fall, front standing fall, back standing fall, left standing fall, and right standing fall) were classified as falls between both low- and high-acceleration ADLs. An insole system that is composed of IMU and FSR sensors is capable of high classification rates for falling events even when other high acceleration ADLs are performed. In addition, by inserting a single insole system into the shoe, the convenience and comfortability to the wearer is improved, thus enabling increased wearer compliance.

## 2. Materials and Methods

### 2.1. Hardware System Overiew

**Figure 1:**
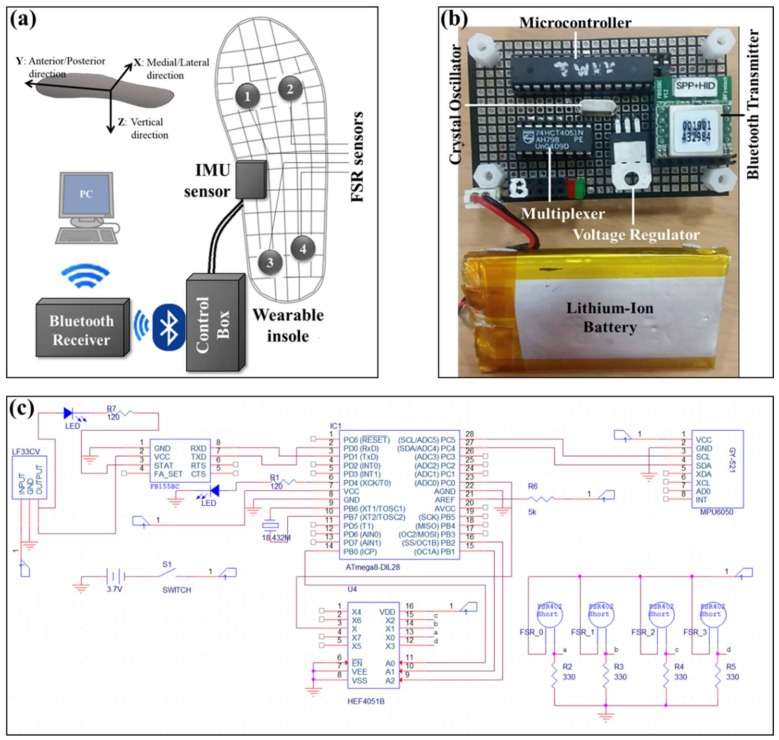
(**a**) Overview of the Hardware System: an insole system comprised of a single Inertial Measurement Units (IMU) and four force-sensitive resistors (FSR) sensors, connected to a control box where a microcontroller calculates sensor measurements, then sends those measurements via Bluetooth to the Bluetooth’s receiver, which collects the streamed data for use in fall detection; (**b**) the contents of the control box; and, (**c**) circuit diagram of the hardware system.

This research constructed an insole hardware system, consisting of an accelerometer from a single IMU (MPU-6050, InvenSense, San Jose, CA, USA) and four FSR sensors (FSR 402 Short, Interlink Electronics, Camarillo, CA, USA). Four FSR, one for each quadrant of the foot, were chosen in an effort to classify falls in the four directions that were performed in this study (forward, backward, lateral-left, and lateral-right). A single FSR, or two FSR’s, which were located at the forefoot, heel, or both, was feared to lack detection power with regards to lateral falls and stumbling falls. The insole system is protected by two sheets of 4 mm thick silicone film, and, using an in-sole template [28], was shaped and sized to fit a size 270 mm male shoe. The IMU includes a tri-axial accelerometer (measuring acceleration with a range of ±8 g). The electronic components in the insole are connected via protective wire to a control box worn above the right ankle and attached via Velcro strap (7 cm × 5 cm × 5 cm, 120 g), inside which a microcontroller (ATMega8A, Atmel, San Jose, CA, USA), in coordination with a multiplexer (HEF4051B, Nexperia, Nijmegen, The Netherlands), allows for the conversion of the four analog FSR resistance values into corresponding digital force values that are then communicated, along with the accelerometer readings, via Bluetooth (FB155BC, Firmtech, Seongnam, Korea) to the Bluetooth’s receiver attached to the computer, as outlined in Figure 1a. Figure 1b details all of the specific components that are contained in the control box, including a microcontroller, 8-channel multiplexer, voltage regulator (LF33CV, STMicroelectronics, Geneva, Switzerland), crystal oscillator, Bluetooth Transmitter, and Lithium-ion Battery. Figure 1c is the circuit diagram of the hardware system.

Data was collected at a sampling frequency of 20 Hz, a frequency that is cited in literature as being sufficiently capable of representing falls and ADLs [15,25]. Data collection at higher frequencies suffers from an increased battery drain and offers minimal or no classification improvement [29]. The microcontroller was programmed using the CodeVisionAVR compiler (CodeVisionAVR 3.27, H.P infotech, New Delhi, India) and a C# program was written to collect the streamed data and store it in a spreadsheet. Matlab was used as the programming language for feature selection and machine learning to classify falls and ADLs.

### 2.2. IMU and FSR Calibration

The IMU orientation was calibrated by laying the insole on a flat surface and observing the associated X, Y, and Z accelerations with respect to gravity, then adjusting the offset [30]. When the IMU is flat and in a static position, a reading of 0 g in the *X*-axis, 0 g in the *Y*-axis, and 1 g in the *Z*-axis should be observed, where *g* = −9.8 m/s^2^. In this research, the FSR are used primarily as an on/off switch or to determine the average force over a period of time. FSR data is computed as the voltage ratio to the zero load condition. Depending on shoe type, foot size, weight, etc., the on/off switch could read non-zero even under conditions where zero load would be expected (lying). As a result, a non-zero on/off switch threshold was experimentally set at 100 from a total 4096 possible analog levels, with 0 equaling 0 V, and 4095 equivalent to 5 V. Measured values of the FSR were especially important to the model in the case of determining the difference between the standing and sitting postures. However, in this research, the calibration of the FSR sensors was deemed to be unnecessary because there is an obvious difference in the FSR values between the standing and sitting postures.

### 2.3. ADL and Falling Experimental Protocol

There are no commonly adhered to standardized protocols for fall detection [26], which results in many unique experimental protocols. This makes it difficult to compare method accuracies between researches in the field [21]. As the focus of this research seeks to distinguish falling events from both low- and high-acceleration ADLs, a new experimental protocol was constructed. The goal in the experimental protocol construction was to include activities in which falls are likely to happen. ADLs and simulated falls should both be safe to the participants and, to the best of the subject’s ability, replicate real-life falls. All of the experiments were performed in accordance with the relevant approved guidelines and regulations of Sungkyunkwan University. All of the participants provided informed consent prior to participating in the experiment. Twenty adult males participants (age: 28 ± 5 years, height: 172 ± 10 cm, weight: 80 ± 17 kg, shoe size: 270 ± 10 mm) participated in an experimental routine that encompassed both low and high-acceleration ADLs and various falling events, as summarized in Table 1 below:

Participants, while wearing their normal footwear, inserted the insole system into their right shoe and completed two minutes of each ADL for a total of 16 min of ADL data per subject. The two minutes of each ADL were then divided into six second windows, for a total of 20 trials of each ADL per subject. In the case of ascending and descending stairs, a staircase consisting of 20 stairs was continuously ascended and descended, with a short pause once the subject reached the top or the bottom of the stairs. The data was then manually split and labeled into their respective stair ascension or descension activity. In addition, each subject performed eight various types of falls, five trials each, on an 8 cm thick mattress cushion, for a total of 40 falls per subject. Deviation between subjects, and even deviation between the trial executions was permitted, as the ideal model should generalize on ADLs and falls performed differently. For example, with regard to falls, some participants braced for impact of the fall with their hands or forearms, while others allowed for their chest, back, or shoulder to first contact the mat. ADLs were also performed differently between trials and subjects. No strict coaching or instruction was given to the subjects. The standing falls consisted of a pre-fall static standing posture, followed by slowly falling over in the given direction. Walking falls were performed by walking approximately a length of 10 m and then falling onto the mat. Walking falls, including a stumble, involved walking approximately a length of 10 m, then stumbling without recovery then falling onto the mat. Running falls and running + stumbling falls were performed similar to the walking falls and walking + stumbling falls, but with increased speed prior to and often during the fall.

### 2.4. Feature Selection and Fall Detection

The ADL and falling data collected at a frequency of 20 samples per second consists of seven measurements in each sample: The acceleration in each axis *X* (medial/lateral), *Y* (anterior/posterior), *Z* (vertical), and each FSR reading (FSR1, FSR2, FSR3, FSR4). These data themselves are not particularly useful, but informative features can be constructed using these inputs that are useful for discriminating falling events from other ADLs.

#### 2.4.1. Accelerometer and FSR Feature Selection

A total of 45 possible features were initially chosen including popular features in activity classification or fall detection research using accelerometers and machine learning and features that show high classification performance. The features were presented in Table 2.

Features 1–24 are common statistical features obtained from an accelerometer. Feature 24 or 25 are features utilized in nearly all of the IMU-based falling detection algorithms; both threshold and machine learning methods. It is called the Sum Vector Magnitude:

$$\mathrm{Sum}\;\mathrm{Vector}\;\mathrm{Magnitude}=\sqrt{Ax^{2}+Ay^{2}\;Az^{2}\;},$$

where *Ax*, *Ay*, and *Az* equal the acceleration in the x, y, and z directions, respectively. It is a feature that is independent of sensor orientation, which is important for identifying falls in different directions or overcoming sensor misalignment [4]. Both the smallest and the largest value in a window were used. During most experimental forward, backward, or lateral falls, the upper body contacts the ground first (shoulder, forearms, chest, back, head), and then the legs and feet raise off the ground in opposition to gravity, followed by the return of the legs and feet to the ground with an assist from gravity. When using an insole sensor, the smallest Sum Vector Magnitude value in this study does not correspond to the initial upper body impact point, but rather the feet. Features 26 and 27 filter the Sum Vector Magnitude signal with a 1st order low-pass Butterworth filer with a 1 Hz cutoff frequency. This specific filter was chosen after a comparison of different order/cutoff frequency combinations. Features 26 and 27 were found to be more informative than features 24 and 25 in distinguishing high acceleration ADLs like running, ascending and descending stairs, and jumping from falling events when using the insole location. The raw feature can be poor at identifying the minimums to create windows when compared to the filtered feature because there is more overlap between falling and high-acceleration ADL’s in the raw feature, as shown in Supplementary Material Figure S1. As a result, feature 26 is a key feature and component of the algorithm, and is used to determine the window’s center. Feature 28 determines the percentage of the window where the filtered Sum Vector Magnitude is below a defined threshold of 0.9. This threshold can separate low-acceleration and high acceleration activities, as well as help to distinguish, in particular, stair descension or ascension, jumping, and falling from one another. Features 30–45 are FSR features that are derived from the on-off switch as well as mean FSR, inspired by gait analysis literature that use FSR features. Features 42–45, in particular, are used to evaluate the end of the window, which could detect if any part of the foot is pressed after a high impact event. This is inspired by, and implemented in a similar fashion as the analysis of accelerometer orientation at the waist after a fall [15,16,31]. Feature 29 measures the variance of the filtered Sum Vector Magnitude signal over the same final two seconds of the window as features 42–45.

#### 2.4.2. Support Vector Machines Falling Detection Algorithm

To overcome the lack of generalizability issues that is associated with threshold models, a machine learning method was created using Support Vector Machines. Support Vector Machines is a supervised learning algorithm for solving classification problems through the creation of a hyperplane that separates data belonging to different categories in a way that maximizes the distance between types [35]. This hyperplane is continually adjusted as new training samples are added to the model to maintain the largest possible distance between class points.

Support Vector Machines is a popular tool used in the fields of activity classification and fall event detection [19]. A multi-classification Support Vector Machines algorithm was designed to distinguish between eight low-/high-acceleration ADLs and falling. It incorporates a polynomial kernel to increase the dimension of the space in order to assist with maximally separating the characteristics of the activities. All of the features were normalized prior to applying Support Vector Machines. The leave-one-out cross-validation method was implemented to evaluate the performance of the model [36]. Leave-one-out cross-validation does not include any data from the test subject in training the model. This process involves the training of a model with 19 of the 20 subjects, and using the 20th subject to test the accuracy of the created model. This procedure is repeated 20-times; leaving out each subject just once as the test subject. The cumulative classification results for all twenty subjects are then compiled to give a description of the overall ADL and the fall classification model.

#### 2.4.3. Genetic Algorithm Process for Feature Selection

Appropriate selection of feature inputs is critical to the success of the machine learning classification algorithms [37]. If all of the 45 features are selected, less descriptive features will be given as much influence on the classification process as the highly important features. Identifying the highly important features and the characteristics from the data allows for an improved falling detection model. A genetic algorithm is a machine learning technique that mimics natural selection to try to identify an optimal solution to a classification problem or feature selection [37]. A genetic algorithm was implemented using Matlab’s function and a random binary gene input of length 45, the same length as the number of possible input features. “1” means that the associated feature is used, while a “0” describes a feature as not used. A simulation (1000 generations, 60 stall generation limit, 0.8 cross-over fraction, 0.01 mutation rate) repetitively seeks to improve the fall classification accuracy by minimizing the fitness function, which was defined as the sum of the falling false positives plus the falling false negatives. True positives exist when a fall occurs and the model identifies a fall. True negatives are when no fall occurs and when the model does not identify a fall. False positives exist when the model declares a fall, but no fall actually occurred, and false negatives take place when a fall occurs, but the model does not detect it. The Genetic Algorithm feature selection was performed in combination with the Support Vector Machines by consecutively evaluating each model’s cross-validation performance. A validation set was preserved from the feature selection process.

#### 2.4.4. Pre-Signal Processing and Window Length for Fall and ADL Classification

A windowing method that was inspired by the windowing method of [18] was utilized. An ADL or falling trial was initially scanned from beginning to end in order to identify the maximum peak Sum Vector Magnitude, as in [18]. The standard maximum and minimum peak Sum Vector Magnitude obtained from the foot location during falls often resemble those features obtained from high-acceleration ADL’s. So instead, from every trial, the smallest filtered Sum Vector Magnitude value was identified, and a window with a 121 frame size is created that is centered on that filtered Sum Vector Magnitude value with 60 frames (3 s) before and 60 frames (3 s) after. Each window that was constructed in this manner is labeled with a number corresponding to the performed activity type. This window’s contents then undergo the statistical and signal analysis that is explained in the Feature Selection section. The window length was evaluated at lengths ranging from 6.05 to 10.05 s, and 6.05 s was determined to be the best performing window length.

### 2.5. Statistical Analysis

The percentage value was analyzed by computing the average and standard deviations. A one-way analysis of variance (ANOVA) test was performed using SPSS 15.0 software (SPSS Inc., Chicago, IL, USA) for analyzing changes in the average percentage of window filtered Sum Vector Magnitude that lies below 0.9 across low-/high-acceleration ADLs and fall. The differences were analyzed using Tukey’s post-hoc test. The statistical significance level was set at 0.01 or 0.05.

## 3. Results and Discussion

### 3.1. Results and Discussion of Feature Selection

A total of 45 features (26 IMU features, 3 new features and 16 FSR features) were considered in this study. A comparison of errors with each feature type and feature combinations are provided in Table 3. Feature numbers correspond to the feature numbers that are listed in Table 2. FP and FN signify False-Positive and False-Negative, respectively.

Current literature uses primarily IMU features to distinguish the falls from low-acceleration ADLs, or high acceleration ADLs [1,4,15,17,18,19]. But, when both high-acceleration ADLs and falls are included, using the Vector Magnitude feature, can completely fail threshold methods [1,4,15] and also confuses and fails machine learning methods [18,19], because high-acceleration ADLs, like jumping, display similar Vector Magnitude peaks and troughs. In this study, when only the IMU features are used, it resulted in (17 FPs and 12 FNs). The primary causes for error were lying postures, high acceleration ADLs stair ascension, stair descension, and especially jumping, which generated extreme acceleration peaks that were similar to falls. In the case of feature selection for IMU features, the selection improved the false positives from 17 FPs to 8 FPs, however it did not improve the 12 FNs. Similar to the non-optimized IMU features, for the optimized IMU features, stair ascension, stair decension, and jumping were the main culprits of misclassification. In particular, falls were too often misclassified as stair descension, resulting in 8 of the 12 FNs. Insole FSR features improve the false negative count, but have higher false positives (27 FPs and 7 FNs), which were struggling to distinguish the difference between low and high-acceleration activities from falling, including: walking, running, standing, stair ascension, and stair descension. This is to be expected, and a reason why traditional fall detection studies prefer to use IMU sensors instead of FSR sensors [4]. There are no false positives or false negatives that are related to the lying or jumping activities, the primary error using only IMU features. Attaching FSR sensors to the insole and using FSR derived features appears to limit errors of those types. In addition, this at least intuitively suggests that IMU and FSR feature types could be combined in a way to improve classification accuracy by helping to cover the ADL classification limitations that are intrinsic to each feature type by itself. A combination of features IMU and FSR resulted in (17 FPs and 9 FNs). This combination type still suffers from the same number of false positives that using only the IMU features does, but it improves the false negative performance. This is an overall accuracy improvement from using features that are only associated with IMU or FSR, because it better covers the full range of low- and high-acceleration ADLs. Finally, the combination of all 45 features, including IMU, FSR, and new features results in (23 FPs and 7 FNs). The high number of false positives is not a desired result. There is no absolute delineation between activities, as every activity except walking and sitting has either false negatives or false positives associated with it. This is likely explained by the inclusion of too many features and decreased relative strength of the most descriptive features [38], necessitating some type of improved feature selection [18,37]. It is through this proper feature selection that the optimal feature combination is found, including selected features from IMU, FSR, and new features. Consequently, the 18 optimal features that are proposed in this study result in (0 FP and 3 FNs).

Table 4 displays the 18 features that were selected that lead to the highest fall detection performance. It includes 14 features that were derived from the accelerometer, and four features derived from the FSR.

**Figure 2:**
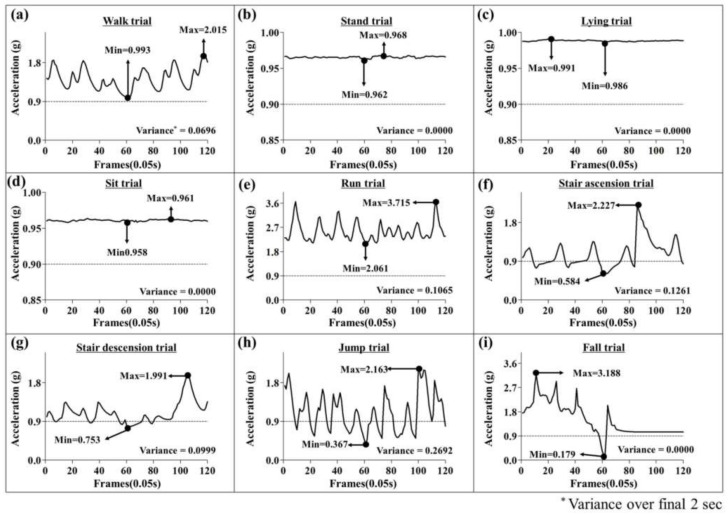
The filtered Sum Vector Magnitude signal from a randomly selected trial for all eight ADLs and a fall. The four low-acceleration ADLs and running all show a minimum filtered Sum Vector Magnitude value above 0.9 (**a**–**e**). High-acceleration ADLs that show a minimum filtered Sum Vector Magnitude value below 0.9 (**f**–**h**). The fall trial’s signal, where the minimum filtered Sum Vector Magnitude is generally lower than that of any other activity (**i**).

**Figure 3:**
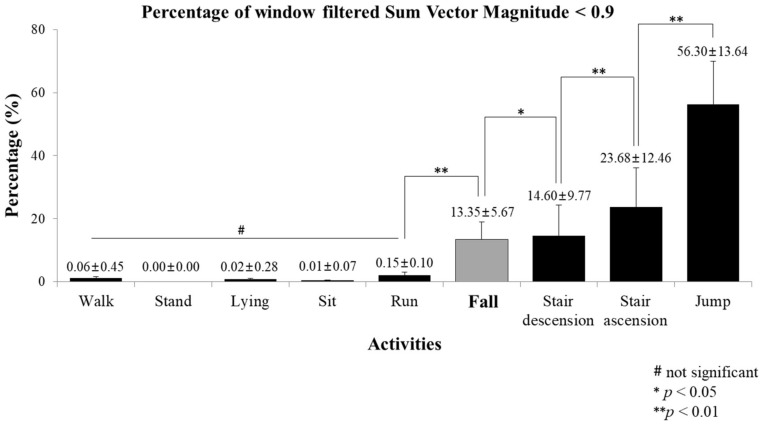
The average percentage of each window where filtered Sum Vector Magnitude is below the 0.9 threshold. This average is computed across all 20 subjects, and all trials, for every ADL and fall.

A single mean acceleration feature was chosen, the mean acceleration in the *Z*-axis, indicating that the mean acceleration in the Z-direction over the course of the window is useful for the differentiation of falls in this model. Both variance and skewness had three features selected, the X, Z and total. This suggests that these features are especially important to the performance of the model. At least one of each of the mean, variance, skewness, kurtosis, and correlation features that were used in [26] were also chosen by this model, indicating that the said features are useful in a machine learning fall detection model. However, zero of the energy related features were chosen. While a useful feature in strictly activity classification research, excluding falling [26], relevant fall classification papers also did not use this feature [18,19]. This is perhaps related to the energy prior to, during, and after a fall can be extremely different, despite all being included in the same window. The minimum filtered Sum Vector Magnitude value is used to center the six second window on the activity, but it is also useful to the model because the minimum filtered Sum Vector Magnitude value that is associated with falling events is frequently lower than that of any other ADL. The maximum filtered Sum Vector Magnitude value was also chosen by the model, and despite running, jumping, stair descension, and stair ascension often generating higher peaks than some falls, this model still could distinguish them, while a threshold would not be suitable. Neither of the traditional minimum Sum Vector Magnitude (feature 24 in Table 2) nor the maximum Sum Vector Magnitude (feature 25 in Table 2) were chosen, which is different from both the threshold and machine learning methods that use the waist attachment location [1,14,16,19,31,39]. This paper contends that, for the foot location, the filtered Sum Vector Magnitude is superior to the traditional Sum Vector Magnitude, as the minimum acceleration peaks that are associated with falling are generally more distinguishable from the peaks that are associated with other high acceleration ADLs. The variance of the filtered Sum Vector Magnitude over the window’s last 2 s was helpful in diagnosing falls, because for falling events, during that portion of the window, the faller was normally lying in a relatively static state with minimal movement. In terms of the FSR features, four features were selected, one of each feature type. One mean FSR feature encompassing the entire window, and one mean FSR feature measuring the window’s final 2 s were used, demonstrating that the mean FSR is important, just like mean accelerations were to the model. Additionally, one on-off switch feature and one FSR duration pressed feature was also selected as being beneficial to the model. The three features that were associated with the FSR located on the heel (33, 36, 40) considered the entire window, while feature 42 measured the mean value of FSR1 over just the window’s final two seconds. The importance of features 26–29 can be explained by referring to Figure 2 and Figure 3 below:

Figure 2 displays the filtered Sum Vector Magnitude signal from a randomly selected trial for each of the nine activities, all being centered on the minimum filtered Sum Vector Magnitude point. Stand, sit, and lying postures, all low-accceleration ADLs, have similar minimum and maximum peak filtered Sum Vector Magnitudes that are above 0.9. The last low-acceleration ADL, walk, has a minimum filtered Sum Vector Magnitude of 0.993, and a maximum of 2.015. All of the high-acceleration ADLs: run, stair ascension, stair descension, jump, as well as the fall trial all have maximum peaks that are ranging from 1.991 to 3.715. The maximum peak is useful to the model for distinguishing the static postures from the non-static postures, but it does not separate the low-acceleration walk, or any of the high acceleration ADLs or falls. The minimum filtered Sum Vector Magnitude values for the first five activities are all above 0.9. These include the four low-acceleration ADLs, plus running. This minimum value is helpful in distinguishing running from other high-acceleration ADLs and falls because for stair ascension, stair descension, jumping, and falling the minimum value goes below the 0.9 threshold, and in the case of this falling trial, to 0.179. The minimum filtered Sum Vector Magnitude value that was obtained from fall trials are regularly lower than the minimum values that are associated with both low-acceleration and high-acceleration ADLs. This is important because the windows are created centered on this value, so if during a fall trial there is a minimum peak that is not associated with the fall itself, but with another high-acceleration ADL, then the model could fail. This is why the traditional minimum Sum Vector Magnitude was not chosen, because the low values that were obtained from high-acceleration ADLs were often lower than the values obtained from falls from the insole location. The variance over the final two seconds is also critical for fall detection. The static activities have a variance of 0.000, whereas walk and the high acceleration ADLs are non-zero. However, because falling events usually culminate in a static lying posture following the fall [14], falls can be distinguished from the other high-acceleration ADLs.

Across all subjects, walk, stand, lying, sit, and run trials have an associated filtered Sum Vector Magnitude that is almost entirely above the 0.9 threshold. However, the stair ascension, stair descension, jump, and fall trials exhibit filtered Sum Vector Magnitude signals that dip below the 0.9 threshold, either multiple times or for extended durations of time. Figure 3 below shows the average percentage of the window that lies below the 0.9 threshold across all of the ADLs and falls for all 20 subjects combined.

Distinguishing falls from high-acceleration ADLs is key to a high performance fall classification system [19]. The low-acceleration ADLs all have a small percentage of the signal that dips below the 0.9 threshold. Run (0.15%), which has a high acceleration that generates high accelerations, rarely dips below 0.9 as well. Meanwhile stair ascension (23.68%), stair descension (14.60%), and jump (56.30%), the other high acceleration ADLs, have a large percentage of the window in which the filtered Sum Vector Magnitude value is below 0.9. Juxtaposing these activities with falls (13.35%), falls exhibit an average percentage that is higher than all low-acceleration ADLs and running, but lower than that of the other high acceleration ADL’s. ANOVA indicated a highly significant (*p* < 0.0001) difference in the average percentage of window filtered Sum Vector Magnitude among low-/high-acceleration ADLs and fall. There was a significant difference in the percentage value between ADLs and fall: Jump > Stair ascension > Stair descension > Fall > Run, Walk, Lying, Sit, Stand (*p* < 0.05 or 0.01). This distinction helps to separate the falling activity from any other activity types, low- or high-acceleration ADLs, so by including this feature the fall classification rate increased. To the best of the author’s knowledge, a similar feature has not been considered by any previous fall classification research. This is likely due to the location of the sensor attachment and because movement of the foot is more pronounced than the hip or chest during high acceleration movements.

### 3.2. SVM Classification and Leave-One-Out Cross-Validation of 8-ADLs and Falls

Table 5 below displays the 9 × 9 confusion table for the ADLs and falling events resulting from the Support Vector Machines model for all twenty subjects, using leave-one-out cross-validation.

In addition to the 9 × 9 classification confusion table, the associated sensitivity, specificity, and accuracy is displayed. Sensitivity is a measurement of the percentage of true positives, or in other words, the likelihood that when a fall occurs the model identifies the fall as a fall. Specificity, which is a measurement of true negatives, is the likelihood that when no fall occurs, the model correctly does not detect a fall. Accuracy is defined as true positives plus true negatives divided by the sum of all the true and false positives and true and false negatives. In total, 797/800 falls were correctly classified as falls, for a sensitivity of 0.996. There were three falls that created false negatives, two of which were misclassified as ascending stairs, and one that was classified as jumping. The three false negatives were all misclassified as high-acceleration ADLs and no falls were misclassified as low-acceleration ADLs, thus confirming that distinguishing high-acceleration ADLs from falls is the most challenging component of fall classification. There were zero false positives, resulting in a specificity of 1.000 and an accuracy of 0.999. These results are comparable to the results of existing fall classification literature using Support Vector Machines. Liu et al. report a sensitivity, specificity, and accuracy of 0.996, 0.997, and 0.991, respectively [19]. Ozdemir et al. obtained a fall sensitivity, specificity, and accuracy of 99.86%, 98.51%, and 99.48%, respectively, and incorporate a larger range of ADL activities, however the system requires the attachment of sensors to six body locations: head, chest, waist, wrist, thigh, and ankle [18].

Of the three false negatives, falls that were misclassified as ADLs, the two that were misclassified as stair ascension activities, were actually a run + stumble type fall, and a back stand fall, while a single standing front fall was misclassified as jump. Again, all three of the false negatives were misclassified as high-acceleration activities. Although a 0.996 fall sensitivity is in line with the results of other representative research in this field, possible explanations for the failed fall detection of the model are presented. The first is with regards to the method that is utilized to create the window. The window is created by locating the lowest filtered Sum Vector Magnitude value, and then taking the previous three seconds and the following three seconds to make the window. But, in the course of a complex falling trial, for example a run fall or a run + stumble fall, there can be other acceleration peaks that, while not likely, can possibly be higher than the fall. This would cause the window for the falling trial to be created centered on a non-fall, which could cause the model to fail by providing it with inaccurate features with respect to falls. Ozdemir et al. created windows in a similar fashion [18], by centering on a Sum Vector Magnitude maximum, so that this explanation would apply to their research as well. A plausible explanation for the back stand fall not being detected is related to the subject that is falling very softly to avoid hurting their head and producing only a very low acceleration with their foot. Literature has demonstrated that the “squat protective response” contributes to these lower peak accelerations during back stand falls [40]. The front fall was misclassified as jumping. This is the result of overlap between the features of the two activity types. Identifying a feature that more clearly distinguishes jumping from falling will be considered in future studies. Lastly, the process of manually labeling experimental data has some labeling error limitations [38], and although every caution and detail was used by the authors, it is still possible that a labeling error occurred, which would lead to decreased performance in the fall detection model.

Zero falling false positives occurred over the course of over 320 total min of ADLs. While some studies have demonstrated sensitivities and specificities that are both as high as 1, they have been proven to have much lower performance in subsequent analysis [4]. This lower performance can be associated with experimental trials being performed differently and the inability for thresholds to be applicable between subjects. It is believed that this machine learning algorithm and insole system overcomes these issues. In addition, these previous studies primarily focus on low-acceleration ADLs. This low-acceleration only design is applicable for a portion of the elderly population, but most falls for the population at large occur during dynamic, high-acceleration activities [22], and this fact requires the creation of models that can distinguish falls from such activities. The elderly population, or even younger people at risk for falls, in order to be healthier, may wish to do regular exercise with associated high-accelerations. Having a sufficient fall detection system in place will give them piece of mind while they exercise or go about their regular days.

A limitation of the study was although 20 male subjects had a shoe size ranging from 260–280 mm, a single 270 mm sized insole was created. This means that the insole did not fit everyone’s foot perfectly, which affected the orientation of the FSR sensors with respect to the foot, in turn affecting the measured force of each FSR. It is conceivable that, if the insole is fit specifically for the subject’s foot, that even higher performance could be obtained. However, it is also of note that FSR sensor calibration has an inherent high error range between sensors [41], so even if a precise, consistent location with respect to the foot is assigned, FSR values will not coincide with FSR measurements on other hardware that is implemented in other studies. Additionally, it is impractical to consider all the possible activities of daily life when creating an experimental procedure, as individuals partake in ADL’s that differ from one another. However, several studies consider transition activities, like sit-to-stand, lie-to-stand, stand-to-sit, stand-to-lie, etc., [18,19]. This study uses the insole location, which is less sensitive to these transition movements than IMU’s that are located at the chest, hip, or waist, and as a result the activities were not part of the experimental protocol. However, properly classifying these transition activities would improve the robustness of the classifier. In future work we plan to expand our activity types to consider transition activities.

## 4. Conclusions

In traditional fall detection literature, fall detection is generally performed using a binary fall detection method—fall, or no fall. This research classifies falls against eight other classes of ADLs, and even classifies the other ADLs as well. Additionally, current literature primarily focuses on differentiating falling from low-acceleration ADLs. This work includes many high-acceleration ADLs, which provide for a more challenging environment to detect high-acceleration falling events. The falling types undertaken were also complex, particularly the non-static falls, and the addition of the “stumbling” component allowed for the subject to effectively create many more than eight types of falls. The selection and utilization of the 18 optimal features offers improved falling classification accuracy when compared to using only IMU, FSR, or all of the features combined. Attaching the insole to the body by inserting it into the shoe is a much more comfortable and attractive solution to the subjects sensing needs when compared to common waist or chest attached locations. The foot location also allows for the utilization of multiple sensors in only one location, as multi-sensor systems can have higher performance than single-sensor systems [25,26], but are generally neglected due to increased discomfort to the user. Future studies seek to increase the types of ADLs and falls in order to train a more robust model. In addition to expanding the ADL types that are performed, testing the model on middle-aged adults or even elderly subjects will also be useful for demonstrating how well the model generalizes between subjects from different age groups.

## Figures and Tables

**Table 1:** The low- and high-acceleration activities of daily life (ADLs) and eight type falls including walking fall, walk and stumble fall, running fall, run and stumble fall, front standing fall, back standing fall, left standing fall, and right standing fall.

	Low-Acceleration ADLs				High-Acceleration ADLs				Falls
Activities	Stand	Lying	Sit	Walk	Run	Stair Ascension	Stair Descension	Jump	Eight fall types

**Table 2:** The 45 available features considered by the fall classification model.

NO	Feature Name	Description	References
1	mean_X	Mean of the *x*-axis acceleration	[18,26]
2	mean_Y	Mean of the *y*-axis acceleration	[18,26]
3	mean_Z	Mean of the *z*-axis acceleration	[18,26]
4	mean_Total	Mean of the total acceleration	[18,26]
5	variance_X	Variance of *x*-axis acceleration	[18,26]
6	variance_Y	Variance of *y*-axis acceleration	[18,26]
7	variance_Z	Variance of *z*-axis acceleration	[18,26]
8	variance_Total	Variance of total acceleration	[18,26]
9	skewness_X	Skewness of *x*-axis acceleration	[18,26]
10	skewness_Y	Skewness of *y*-axis acceleration	[18,26]
11	skewness_Z	Skewness of *z*-axis acceleration	[18,26]
12	skewness_Total	Skewness of total acceleration	[18,26]
13	kurtosis_X	Kurtosis of *x*-axis acceleration	[18,26]
14	kurtosis_Y	Kurtosis of *y*-axis acceleration	[18,26]
15	kurtosis_Z	Kurtosis of *z*-axis acceleration	[18,26]
16	kurtosis_Total	Kurtosis of total acceleration	[18,26]
17	energy_X	Energy of *x*-axis acceleration	[26]
18	energy_Y	Energy of *y*-axis acceleration	[26]
19	energy_Z	Energy of *z*-axis acceleration	[26]
20	energy_Total	Energy of total acceleration	[26]
21	correlationX_Y	Correlation between *x*- and *y*-axes acceleration	[26]
22	correlationX_Z	Correlation between *x*- and *z*-axes acceleration	[26]
23	correlationY_Z	Correlation between *y*- and *z*-axes acceleration	[26]
24	min Sum Vector Magnitude	Minimum Sum Vector Magnitude value	[1,14,18,19,31]
25	max Sum Vector Magnitude	Maximum Sum Vector Magnitude value	[1,14,18,19,31]
26	min filtered Sum Vector Magnitude	Minimum low-pass filtered Sum Vector Magnitude value	New Feature
27	max filtered Sum Vector Magnitude	Maximum low-pass filtered Sum Vector Magnitude value	New Feature
28	filtered Sum Vector Magnitude < 0.9 duration	Percent of window where the low-pass filtered Sum Vector Magnitude value is less than 0.9	New Feature
29	variance filtered Sum Vector Magnitude over window’s final 2s	Variance of the filtered Sum Vector Magnitude over final two seconds in the window duration	[18,26]
30	FSR1 switch on duration	Percent of the window where FSR1 is on	[24,32]
31	FSR2 switch on duration	Percent of the window where FSR2 is on	[24,32]
32	FSR3 switch on duration	Percent of the window where FSR3 is on	[24,32]
33	FSR4 switch on duration	Percent of the window where FSR4 is on	[24,32]
34	FSR1 total on-off switches	Total number of times FSR1 readings switch on or switch off	[32]
35	FSR2 total on-off switches	Total number of times FSR2 readings switch on or switch off	[32]
36	FSR3 total on-off switches	Total number of times FSR3 readings switch on or switch off	[32]
37	FSR4 total on-off switches	Total number of times FSR4 readings switch on or switch off	[32]
38	mean_FSR1	Mean FSR1 value over window duration	[33,34]
39	mean_FSR2	Mean FSR2 value over window duration	[33,34]
40	mean_FSR3	Mean FSR3 value over window duration	[33,34]
41	mean_FSR4	Mean FSR4 value over window duration	[33,34]
42	mean_FSR1 over window’s final 2s	Mean FSR1 value over final two seconds in the window duration	[33,34]
43	mean_FSR2 over window’s final 2s	Mean FSR2 value over final two seconds in the window duration	[33,34]
44	mean_FSR3 over window’s final 2s	Mean FSR3 value over final two seconds in the window duration	[33,34]
45	mean_FSR4 over window’s final 2s	Mean FSR4 value over final two seconds in the window duration	[33,34]

**Table 3:** Comparison of errors based on feature combinations.

Feature Type	Feature Number	Error
IMU features	#1~#25, #29	17 FPs and 12 FNs
Optimized IMU features	#3, #7–9, #12, #16, #21–22, #24	8FPs and 12FNs
FSR features	#30~#45	27 FPs and 7 FNs
IMU and FSR features	#1~#25, #29~#45	17 FPs and 9 FNs
All features	#1~#45	23 FPs and 7 FNs
Optimal features (used in this study)	#3, #5, #7–9, #11–13, #16, #22, #26–29, #33, #36, #40, #42	0 FP and 3 FNs

**Table 4:** Selected features for optimal fall detection.

No	Selected Feature Name	Feature Type	References
3	mean_Z	from Accelerometer	[18,26]
5	variance_X		[18,26]
7	variance_Z		[18,26]
8	variance_Total		[18,26]
9	skewness_X		[18,26]
11	skewness_Z		[18,26]
12	skewness_Total		[18,26]
13	kutosis_X		[18,26]
16	kurtosis_Total		[18,26]
22	correlationX_Z		[26]
26	Minimum filtered Sum Vector Magnitude		New feature
27	Maximum filtered Sum Vector Magnitude		New feature
28	filtered Sum Vector Magnitude < 0.9 duration		New feature
29	Variance filtered Sum Vector Magnitude over window’s final 2 s		[18,26]
33	FSR4 switch on duration	from FSR	[24,32]
36	FSR3 total on-off switches		[32]
40	Mean_FSR3		[33,34]
42	Mean_FSR1 over window’s final 2 s		[33,34]

**Table 5:** 9 × 9 confusion Table for low-, high-acceleration ADLs and fall classification.

	Low-Acceleration ADLs				High-Acceleration ADLs				Falls		
	Walk	Stand	Lie	Sit	Run	Stair Ascension	Stair Descension	Jump	Falls	Total	Sensitivity
Walk	382	1	0	0	14	1	2	0	0	400	**0.955**
Stand	3	369	1	27	0	0	0	0	0	400	**0.923**
Lie	0	3	397	0	0	0	0	0	0	400	**0.993**
Sit	2	53	0	343	0	0	2	0	0	400	**0.858**
Run	38	0	0	0	362	0	0	0	0	400	**0.905**
Stair Ascension	2	0	0	0	1	349	48	0	0	400	**0.873**
Stair Descension	0	0	0	0	2	56	340	2	0	400	**0.850**
Jump	0	0	8	0	0	6	11	375	0	400	**0.938**
Fall	0	0	0	0	0	2	0	1	797	800	**0.996**
Total	427	426	406	370	379	414	403	378	797		
**Specificity**	**0.992**	**0.995**	**0.983**	**0.997**	**0.987**	**0.981**	**0.982**	**0.999**	**1.000**		
**Accuracy**	**0.978**	**0.985**	**0.977**	**0.997**	**0.983**	**0.970**	**0.968**	**0.993**	**0.999**		

## Supplementary Materials

The following are available online at http://www.mdpi.com/1424-8220/18/4/1227/s1, Figure S1: Comparison of features 24 and 26 across all experimental trials.
