# Supplementary material for: A Novel Detection Model and Its Optimal Features to Classify Falls from Low- and High-Acceleration Activities of Daily Life Using an Insole Sensor System
Source: Sensors (Basel). 2018 Apr 17;18(4):1227. doi: 10.3390/s18041227 (PMC5948845; doi:10.3390/s18041227)
Supplement: Supplementary file 1 [file sensors-18-01227-s001.pdf]

**Supplementary Material**

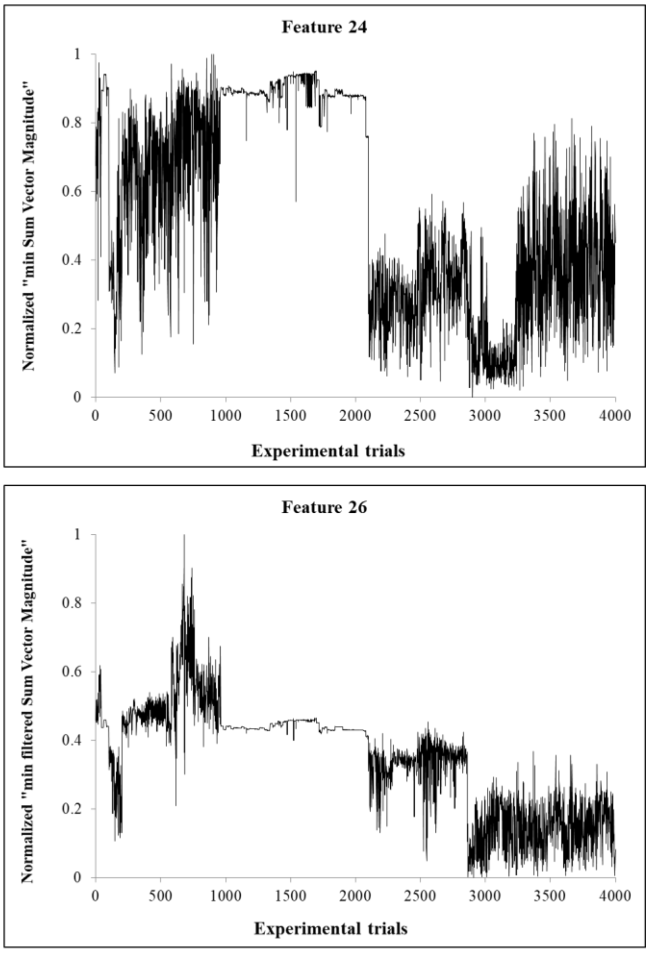


**1-400 : Walk**
**401-800 : Run**
**801-1200 : Stand**
**1201-1600 : Lying**
**1601-2000 : Sit**
**2001-2400 : Upstairs**
**2401-2800 : Downstairs**
**2801-3200 : Jump**
**3201-4000 : Falling**

**Figure S1.** Comparison of features 24 and 26 across all experimental trials.
